# Enhancing translational research in metastatic cancer through an open science environment: the UPTIDER experience

**DOI:** 10.1038/s41698-025-01110-5

**Published:** 2025-11-06

**Authors:** Anirudh Pabba, Marion Maetens, Amena Mahdami, Tatjana Geukens, Maxim De Schepper, Gitte Zels, Karen Van Baelen, Sophia Leduc, Ha Linh Nguyen, Josephine Van Cauwenberge, Kristien Borremans, Sigrid Hatse, Madita Nysen, Emma Rousseau, Angelica Di Tommaso, Bram Boeckx, Patrick Neven, Hans Wildiers, Elia Biganzoli, Peter Vermeulen, Wouter Van Den Bogaert, Diether Lambrechts, Giuseppe Floris, Christine Desmedt, François Richard

**Affiliations:** 1 Laboratory for Translational Breast Cancer Research, Department of Oncology, KU Leuven, Leuven, Belgium; 2 General Medical Oncology Unit, Department of Oncology, UZ Leuven, Leuven, Belgium; 3 Department of Pathology, UZ Leuven, Leuven, Belgium; 4 Gynecological Oncology Unit, Department of Oncology, UZ Leuven, Leuven, Belgium; 5 Laboratory of Experimental Oncology (LEO), Department of Oncology, KU Leuven, Leuven, Belgium; 6 Laboratory for Translational Genetics, Department of Human Genetics, KU Leuven, Leuven, Belgium; 7 Unit of Clinical Research and Medical Statistics, Department of Biomedical and Clinical Sciences (DIBIC) & DSRC, LITA Vialba campus, University of Milan, Milan, Italy; 8 Translational Cancer Research Unit, Oncology Center Antwerp, Ziekenhuis aan de Stroom, Antwerp, Belgium; 9 Department of Forensic Medicine, University Hospitals Leuven, Leuven, Belgium; 10 Center for Cancer Biology, VIB, Leuven, Belgium; 11 Laboratory for Translational Cell and Tissue Research, Department of Pathology and Imaging, KU Leuven, Leuven, Belgium

**Keywords:** Computational biology and bioinformatics, Health care, Medical research

## Abstract

Translational research in metastatic cancer is limited by insufficient metastatic samples. Post-mortem tissue donation programs address this issue by facilitating comprehensive sample collection. Sustaining such programs requires an open science environment (OSE) to ensure multidisciplinary collaboration, research standards, and patient privacy. While often seen at publication, we demonstrate the benefit of developing upstream phases by presenting the OSE from our institutional post-mortem tissue donation program UPTIDER (NCT04531696). It contains (i) an electronic case report form to capture >750 clinical features including treatment lines and metastases, (ii) a lab information management system to track >100 metadata features from logistical to anatomical information, (iii) a code versioning system, (iv) long-term data and sample storage, and (v) code and data sharing upon publication. By ensuring latest access to information, our OSE reflects the potential to accelerate translational research. While our OSE was tailored for UPTIDER, we believe our experiences can inspire others.

## Introduction

Open science is a set of principles and practices that promote transparency and collaboration in scientific research^1–3^. These include accessibility, interoperability, reproducibility, and accountability^1,2,4,5^. This promotes research continuity, peer validation, research progress and communication^2,4,6^. While beneficial when implemented at study conception, they are often only partly implemented during study publication. This has implications, particularly for translational research, where teams composed of multidisciplinary personnel, require continuous access to the latest and most accurate data. An open science environment (OSE), defined here as a network of tools that support open science, can fulfil this need. An OSE incorporates FAIR principles (Findable, Accessible, Interoperable and Reusable)^7^ and a data management plan (DMP)^8^. The OSE includes comprehensive study designs, implementation of research data management (RDM) systems and computational tools built using globally accepted standards^3^. It incorporates key research elements including data reporting using persistent identifiers (PID), metadata annotation, code versioning, protocol documentation and data storage management^6,9,10^. Data can be deposited in data repositories^11^ while code along with processed data, can be deposited in code capsules according to the “as open as possible, but as closed as necessary” principle in data sharing^7,12^. These datasets are then published with an official digital object identifier (DOI) under specific licensing^3^. This practice has already been shown to boost citation impact^13,14^.

According to the Budapest-Bethesda-Berlin definition, Open access (OA) publishing ensures unrestricted and free public access to research publications^15,16^. It is classified into five categories, where gold and diamond OA have the highest article processing charges (APC) and access rights^15^. In addition, community-led preprint servers such as BioRxiv or MedRxiv and initiatives such as Peer Community In (PCI) and Open Research Europe, have emerged as free alternatives to bring research publications directly to peers, while eliminating prolonged delays often seen with academic journals^17,18^. However, challenges such as insufficient funds, guidance, community support, training opportunities and institutional encouragement hinder adoption of these principles^5,19^.

Open science has seen implementation in animal research^20^ and human sociological research^21^, but its application in human translational research remains unclear due to the lack of consensus on open science practices and logistical support^22^. This creates a knowledge gap, as human translational research is bound by legal regulatory frameworks such as the General Data Protection Regulation (GDPR) in Europe^23^, and the Health Insurance Portability and Accountability Act (HIPAA) in the United States of America^24^. These frameworks promote responsible research by implementing privacy protections, including pseudonymizing patient information. While this has presented certain challenges for cross-border data sharing^25^, compliance with these frameworks within an OSE ensures sustainable research, and data reuse^26–28^.

Cancer incidence remains a ubiquitous challenge for public health^29^. While massive data repositories for patients with primary cancer exist^26^, limited data on metastatic disease restricts opportunities for improved disease management strategies. This is an unmet need considering metastatic disease is one of the leading causes of cancer mortality^30^. Post-mortem tissue donation programmes overcome this challenge by generating many samples, at various timepoints while integrating transdisciplinary collaboration^31^. This, however, requires a multi-layered approach to document, annotate, analyse and share clinical and sample metadata in a GDPR and FAIR compliant environment, like an OSE. Considering the challenges in developing an OSE, we present our experiences in the context of our institutional programme UPTIDER (UZ/KU Leuven Post-mortem Tissue Donation to Enhance Research, NCT04531696)^32^ to advance translational research using samples and data from patients with metastatic breast cancer.

## Results

### UPTIDER, a highly collaborative project requiring a robust OSE

**Fig. 1:**
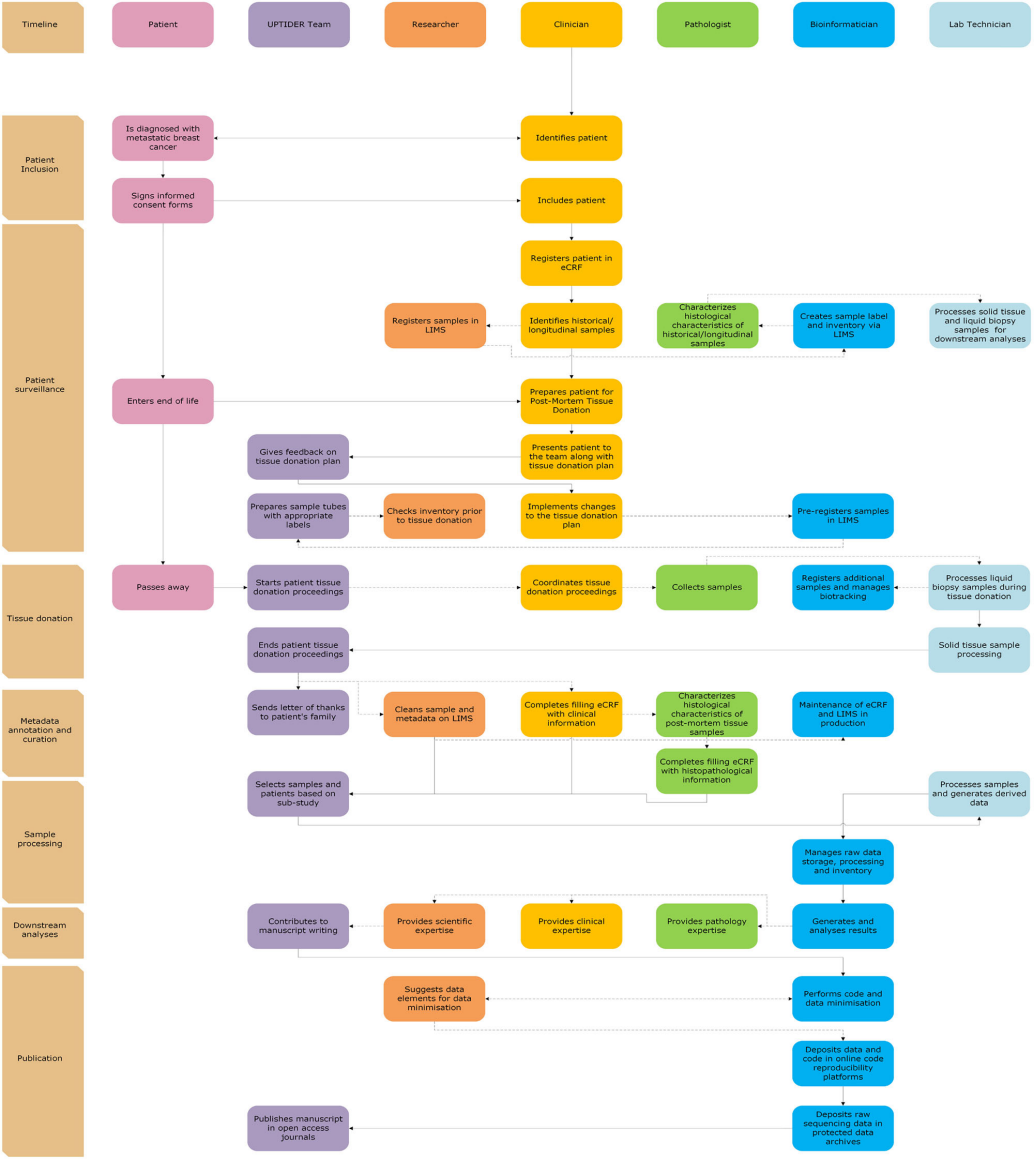
The UPTIDER process from patient inclusion to study publication. A flowchart and timeline describe the key processes within UPTIDER attributed to each of the players in the team from patient inclusion to study publication. The flowchart elucidates the degree of inter-player communication and collaboration within the UPTIDER team, emphasizing the logistical and scientific need for a UPTIDER OSE to support its research objectives. The timeline outlines the major events between patient inclusion and study publication. Rectangles in pink describe the patient trajectory within the OSE, purple describes the actions performed by the entire team, followed by actions of the researcher, clinician, pathologist, bioinformatician and lab technician in orange, yellow, light green, blue and light blue rectangles, respectively. The role of a researcher can be embodied by a clinician, lab technician, pathologist, scientist and/or research data manager. Solid arrows indicate dependent relationships between actions, where the previous action must end before the next one can start. Dashed lines indicate a parallel flow to the main action. Not displayed on the figure, but during tissue donation, samples are also processed for external institutional collaborators. Additionally, data and material sharing are facilitated after approval of the ethics committee and the signing of material and data transfer agreements. These agreements are drafted in collaboration with the KU Leuven legal department. For access to raw sequencing data, a request needs to be made to the data access committee via the EGA portal. Extended version of the flowchart is available in Supplementary Fig. S1. eCRF: electronic case report form, LIMS: lab information management system, OSE: open science environment, UPTIDER: UZ/KU Leuven Post-mortem Tissue Donation to Enhance Research. Created with Microsoft Visio.

**Fig. 2:**
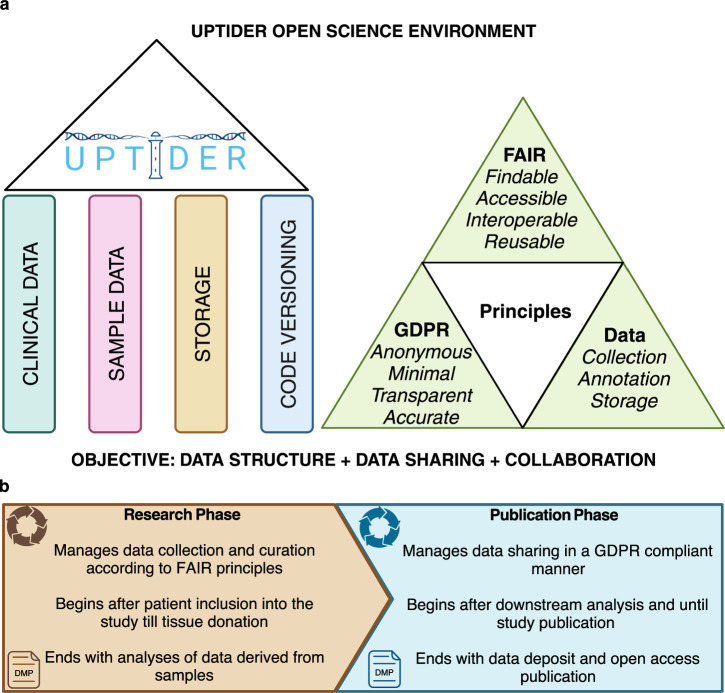
Structure and guiding principles of the UPTIDER OSE. **a** Graphical abstract describing the 4 main structural pillars used to design the components of the UPTIDER OSE, followed by the key guiding principles which were incorporated into the design. These enabled the OSE to satisfy the key objective of the UPTIDER OSE, which was to provide a robust data structure during data collection, annotation and storage while making data sharing convenient and collaboration feasible. **b** Graphical abstract describing the two principal phases within the UPTIDER OSE during each research lifecycle. While these phases are linked by a linear flow from one to the other, each of these phases were also independently governed and supported by the overarching DMP. This created an independent provision for continuous development and improvement to both phases throughout the research lifecycle, making the OSE an adaptive entity. Created with biorender.com. DMP: data management plan, OSE: open science environment, UPTIDER: UZ/KU Leuven Programme for post-mortem Tissue Donation to Enhance Research.

**Fig. 3:**
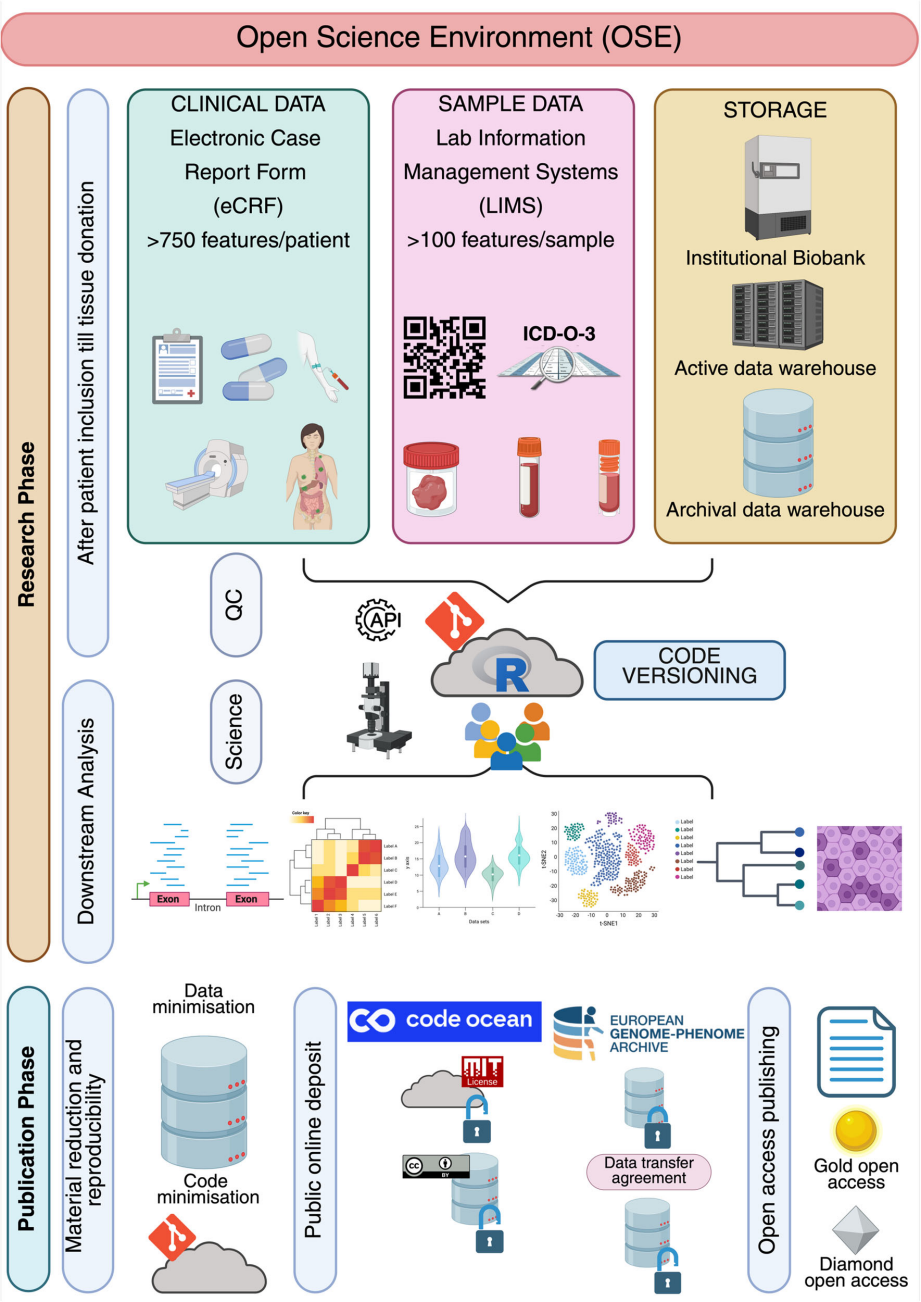
The components of the UPTIDER open science environment. A description of the structural pillars and phases of the UPTIDER OSE along with their features. At the start of the research lifecycle, patients are included into UPTIDER. Clinical data, including more than 750 clinical features, are collected per patient using an eCRF in line with GDPR principles. Sample metadata, including more than 100 features per sample, are collected using a LIMS in line with FAIR principles. Sample data and sample generated data are stored in the institutional biobank and GDPR protected data warehouses that comply with regulatory frameworks. Following tissue donation, data curation and generation, quality checks and downstream analyses are performed for various UPTIDER sub-studies using the distributed code versioning system. Once research manuscript is sent for publication, material reduction (code and data) is performed in compliance with GDPR principles for data sharing and reproducibility, meaning that only the code and data essential to reproduce the study are shared. This material is then deposited into secure online public access environments such as Code Ocean under appropriate licensing for data re-use and reproducibility. The raw data is deposited into European Genome-Phenome Archive under restricted access, which can be requested following a data transfer agreement. Similarly sample access can be requested following a material transfer agreement. In parallel, the research manuscript undergoes peer-review and gets published in either a gold or a diamond open-access journal. GDPR: general data protection regulation, UPTIDER: UZ/KU Leuven Programme for post-mortem Tissue Donation to Enhance Research. Created with biorender.com.

UPTIDER is a highly collaborative programme intended to enhance our understanding of metastatic breast cancer^32^. Figure 1 and Supplementary Fig. S1 describe the landmark events within UPTIDER highlighting both the required intra-team collaboration and the importance of providing the team with prompt access to latest information. This also explains the logistical and scientific need for developing and maintaining an OSE in UPTIDER (Fig. 2 and Methods). In this regard, as of May 2025, the UPTIDER OSE helped facilitate the acquisition and annotation of >15000 samples from 39/45 enroled patients. These samples were acquired from >30 sites of solid tissue and 7 distinct sources of liquid biopsy leading to a median of 300 samples per autopsy. Figure 3 provides a global overview of the components of the OSE and their trajectory based on an established framework design and timeline, illustrating the foundation required to implement open science practices within UPTIDER. Complementing Fig. 3, Supplementary Fig. S2 presents the architectur diagram of the OSE highlighting data and code flows between user, storage, and computational resources.

### Capturing clinical data

**Fig. 4:**
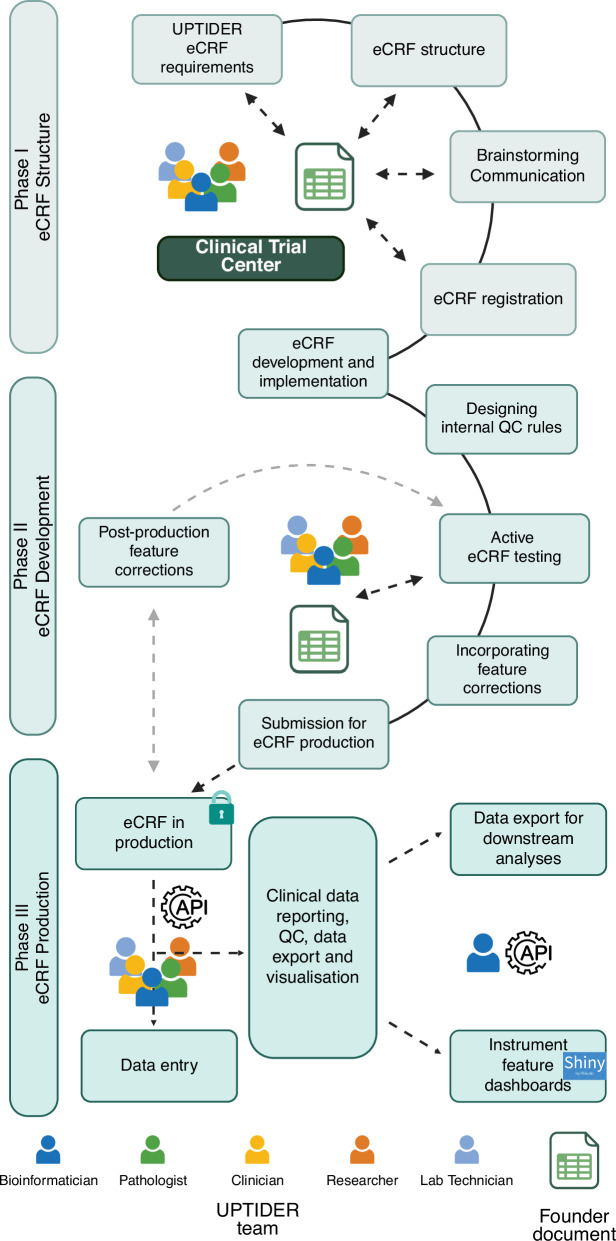
Envisioning and implementing the UPTIDER eCRF. A brief description of the methodology employed to design and implement the UPTIDER eCRF as a sequential process. The methodology involved 3 different phases linked to the eCRF structure, development and production and ultimately the final design had to comply with the GDPR principles. Constant communication between the UPTIDER team, and the clinical trial centre facilitated the development of the founder document and the preliminary registration of the eCRF emphasized by the double-tailed arrows. During development, internal QC rules and clinical features were designed, tested and implemented using the founder document in communication with the UPTIDER team. Once the eCRF was submitted for production, post-production feature corrections were kept to a minimum, highlighted by the grey-dashed arrows, to prevent data corruption and loss during data entry. Data entry was facilitated by both manual entry with the team and with SQL query via API. This API also helped in the data export of the eCRF to be used for data curation, visualization and reporting. API: application programming interface, eCRF: electronic case report form, GDPR: general data protection regulation, SQL: structured query language, UPTIDER: UZ/KU Leuven Programme for post-mortem Tissue Donation to Enhance Research. Created with biorender.com. Schematic based on REDCAP (v15.0.37).

To improve clinical outcomes of future patients by translating current fundamental research into the clinic, up-to-date access to patient electronic health record (EHR) is needed to design research objectives and derive meaningful conclusions from patient data^33,34^. As such, patients with metastatic cancer are treated with multiple treatment regimens, undergo multiple blood draws and biopsies, as well as several surgeries and imaging examinations throughout their disease course. This means that each patient comes with a complex medical and clinical history, which needs to be combined with sample data to perform translational research. To satisfy this requirement and to enable protected access to data for the entire team, an electronic case record form (eCRF) needed to be designed. The first phase was to identify pivotal clinical features from the patient EHR for our study requirements and then develop a structure using a founder document (Fig. 3, Fig. 4 Phase I and Supplementary Table S1). This was an independent document that co-existed alongside the eCRF. The key purpose of this document was to capture proceedings from the brainstorming and design sessions for features that were to be included. After consensus was achieved on the feature structure from the founder document, an eCRF was registered. A second phase was then initiated to design and implement this structure into the eCRF (Fig. 4, Phase II). Constant collaboration with the clinical trial centre (CTC) at UZ Leuven was crucial in helping refine our requirements and feature structure before efficiently translating them into an eCRF. Once the eCRF was designed, internal quality checks were added to ensure accurate data input. We developed more than 25 internal quality checks (QC) by adding branching logic and data validation to specific fields, preventing inconsistent information. Instruments resembled a survey environment with structured drop-down menus and multiple-choice answers to ease data recording and to reduce missing data. Helper texts were added to increase consistent reporting. Predefined missing codes were implemented to capture reason of missing data to identify potential bias in downstream analyses. Additionally, free-text fields were minimized to reduce unstructured information. For features requiring recurrent updates such as team member and treatment regimens, structured query language (SQL) queries were developed to ease item addition into pre-existing dropdown lists. Once implemented, the eCRF was thoroughly tested by the entire team. Here, the founder document was instrumental in communicating with the UPTIDER team to capture feedback and track progress (Fig. 4, Supplementary Table S1). Post-validation, the eCRF was submitted for production (Fig. 4, phase III), allowing the team to record patient EHR data (Fig. 2). At this stage, modifications to the eCRF had to be as minimal as possible, since all modifications must be approved by the institution while ensuring no data loss. Eventually, more than 750 unique features per patient were recorded in the eCRF (Fig. 3) to capture clinical and autopsy data. The codebook of the UPTIDER eCRF can be found in the Supplementary Table S2. During data entry, each participating patient was encoded with a unique ID that was used to identify and annotate each feature in the patient record. The structure of the eCRF together with an application programming interface (API) allowed for data entry, export and reporting (Fig. 4, Phase III). The provision of role-based access and multifactor authentication (MFA) selectively enabled the team to securely record authorised clinical and non-clinical information in accordance with GDPR principles. Taken together, we have demonstrated the value of inter-team collaboration and highlight the extensive processes involved in the conception, development, testing, and production of the eCRF.

### Recording sample data and metadata

**Fig. 5:**
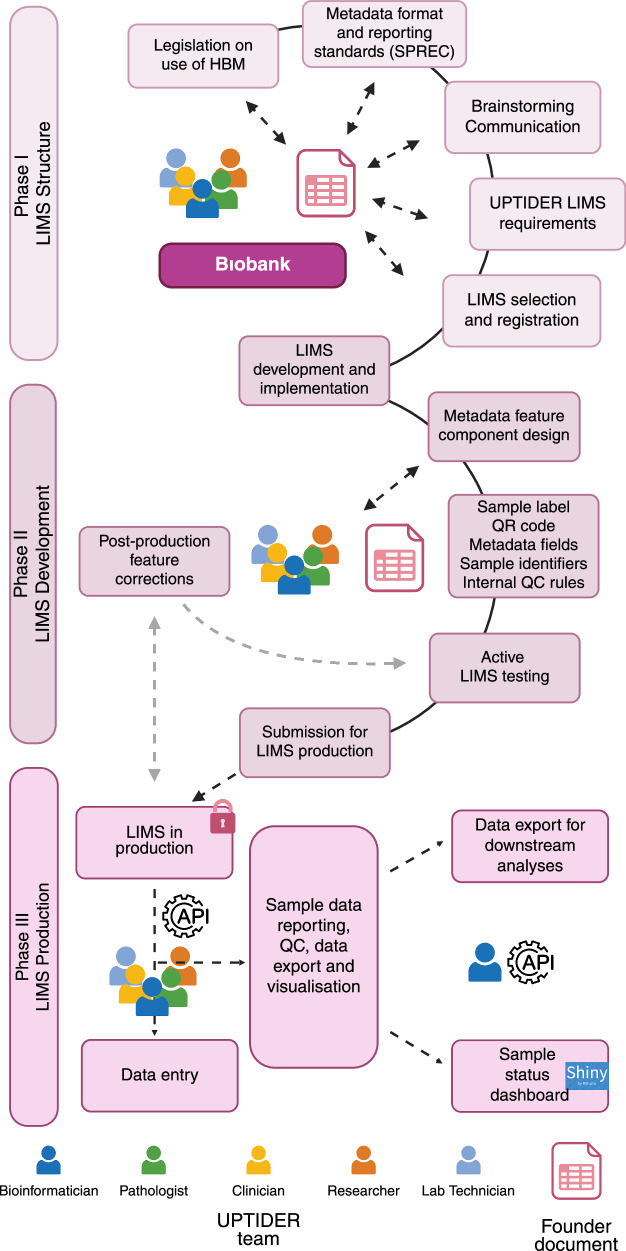
Envisioning and implementing the UPTIDER LIMS. A brief description of the methodology employed to design and implement the UPTIDER LIMS as a sequential process. The methodology involved 3 different phases linked to the LIMS structure, development and production and the final design had to comply with the regulatory legislation, SPREC reporting standards for metadata and FAIR principles. Constant communication between the UPTIDER team, and the institutional biobank facilitated the development of the founder document and the selection of the LIMS emphasized by the double-tailed arrows. During development, internal QC rules and metadata features were designed, tested and implemented using the founder document in communication with the UPTIDER team. Once the LIMS was submitted for production, post-production feature corrections were kept to a minimum, highlighted by the grey-dashed arrows, to prevent data corruption and loss during data entry. Data entry was facilitated by an SQL query via API. This API also helped in data export of the LIMS to be used for data curation, visualization and reporting. API: application programming interface, FAIR: findable, accessible, interoperable and reusable, LIMS: lab information management system, SPREC: standard preanalytical code, SQL: structured query language, UPTIDER: UZ/KU Leuven Programme for post-mortem Tissue Donation to Enhance Research. Created with biorender.com. Schematic based on Labcollector (v6.262).

An UPTIDER autopsy can yield up to 600 solid and liquid samples^32^. This necessitates a robust data and metadata structure, that can be routinely updated by the team after using various research techniques on these samples (Fig. 1). We therefore customised our lab information management system (LIMS) by initiating an appropriate feature structure design (Fig. 5, Phase I). The structure needed to accommodate the legal requirement for the use of human body materials (HBM), standard preanalytical coding for biospecimens (SPREC) metadata reporting standards and institutional biobank recommendations (Supplementary Table S3), while also satisfying the internal metadata needs of UPTIDER. At this stage, collaboration and correspondence with the biobank was crucial. Like the eCRF, this structuring phase was supported by a founder document (Supplementary Table S3), which registered metadata design sessions and intra-team communication. In UPTIDER, wherever possible, a tissue site is sampled under multiple conditions in mirror (formalin-fixed paraffin embedded (FFPE), Fresh frozen in optimal cut temperature medium (FF-OCT) and fresh frozen (FF) for instance) for various analyses (Fig. 3). FFPE allows histopathological assessment for essential tumour characterization, while FF enables next-generation sequencing (NGS) of DNA and/or RNA among others. Therefore, information from FFPE samples must be linked to FF samples used for sequencing, which lab technicians extract and bioinformaticians analyze. Thus, mirrored sample linkage was a mandatory requirement within the LIMS. Another fundamental requirement was the generation and linkage of derived samples such as DNA libraries that originate from a parent sample (FFPE, FF-OCT or FF). Selecting a suitable LIMS to fulfil these requirements while being financially sustainable was crucial. As an institutional LIMS was not available, we explored different players in the market. Post-identification and feature approval, we initiated the LIMS development using the founder document (Fig. 5, Phase II). At this stage, we added approved metadata feature components into the LIMS and included a unique serial ID for each patient, complementing the LIMS identifier. This created an internal double authentication system, securing sample identification and tracking. Internal QCs were also designed to prevent incorrect data entry (Fig. 5). Additionally, active testing of the LIMS was facilitated by the founder document with documentation of feature corrections from the team along with a status tracker. We then initiated the final phase of the LIMS and submitted our LIMS for production (Fig. 5, Phase III). Here, data entry was largely supported by automated code scripts. As a result, more than 100 features per sample have been implemented including sample label, date of collection and organ code according to the ICD-O-3 organ classification^35^ (Supplementary Fig. S3). Mirrored and derived samples are automatically handled with a R script using the API. An audit trail logs user activity across samples. Each sample is given a label with a QR code and structured text to guide sample collection (Supplementary Fig. S3). This QR code is linked to the LIMS ID and helps keep track of the sample throughout its lifecycle. Additionally, to avoid data loss, samples registered but not collected are never deleted but rather, archived. A version of the LIMS codebook can be found in Supplementary Table S4. The LIMS and the eCRF comply with FAIR principles and are linked by the patient ID. Electronic lab notebooks (ELN) are used to document and track protocols for the sample processing. Each notebook is assigned to a specific protocol with version numbers and a timeline to trace the eventual development of the standard operating protocol (SOP)^32^. Taking these into consideration, we report on the collaborative efforts on designing the appropriate methodology for our custom metadata structures and selecting a LIMS that could accommodate legal requirements and internal needs while ensuring accurate sample tracking.

### Identifying sources for sample and data storage

For long-term usage and sharing of UPTIDER samples, appropriate storage and inventory solutions needed to comply with GDPR and FAIR principles while supporting automated data annotation. All samples collected during tissue donation have been securely stored at the biobank under restricted access. Our LIMS supports bio-tracking by registering sample storage locations and is continuously improved to maintain accuracy in metadata reporting. Additionally, the LIMS metadata structure aligns with biobank requirements, enabling automated metadata sharing and compatibility with initiatives like the new European Health Data Space for enhanced public health research^36^.

**Fig. 6:**
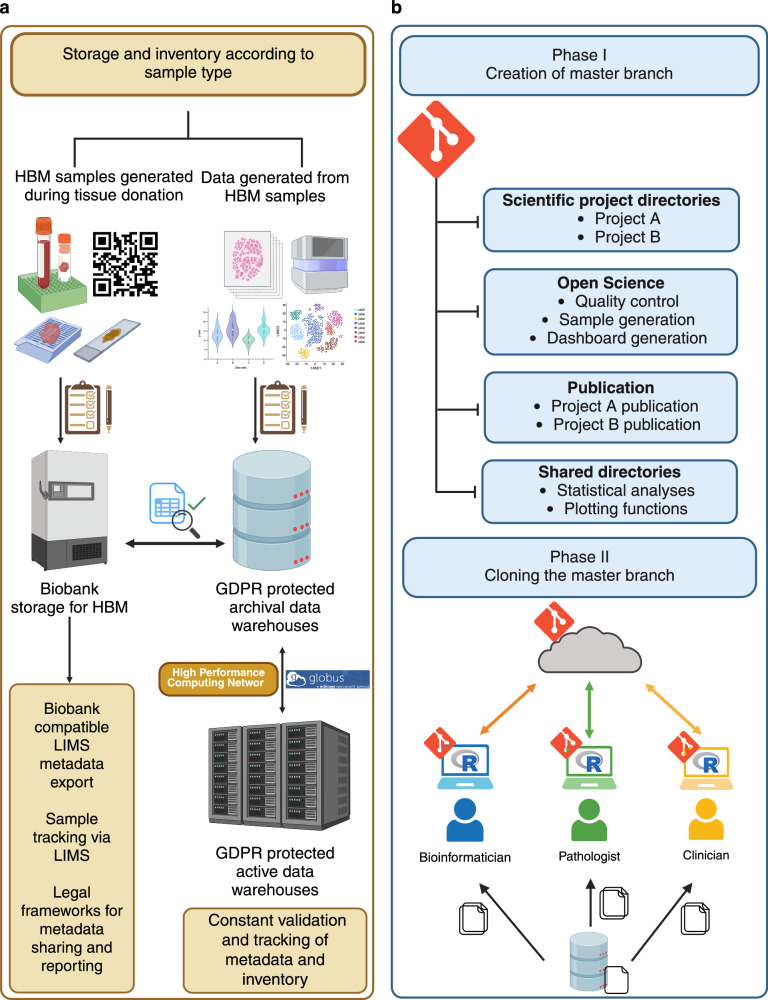
Identifying storage solutions for UPTIDER data and establishing a distributed code versioning system. **a** Graphical description regarding the selection of storage solutions to store UPTIDER sample data and generated data. For samples collected after patient inclusion into UPTIDER, inventory is performed and stored at the institutional biobank with bio-tracking facilitated by the UPTIDER LIMS. The LIMS metadata structure is compatible with the regulatory requirements of the biobank for use of HBM, while making data sharing, depositing and retrieval convenient. For data and results generated from these samples with various research technologies, inventory is performed, and they are stored in GDPR protected archival data warehouses. A live excel 365 document is used to couple information from samples sent for NGS with their corresponding LIMS metadata. High-performance computing networks are used to transfer and process these sequencing files between the network and the archival warehouse. For frequently used data, active data warehouses and high-performance computing networks are used to improve data transfer speeds in place of archival warehouses. To facilitate this transfer and to track the location of the data, warehouse workflow managers are used. **b** Graphical description regarding the design and implementation of the distributed code versioning system. The master branch of the versioning system maintained by the lead bioinformatician is designed to host types of code scripts linked to scientific projects, maintaining open science standards, minimized code for publication and common code for shared project directories. The code scripts also have pre-defined code calls to access data prior to processing. This code call is designed to mimic the data hierarchy and structure of the archival data warehouse. The branch is then cloned to other members of the team, after which personnel can either begin replicating the code for their purposes or make new code scripts per requirement, which are then logged, committed and pushed to the master branch to be pulled and reproduced by other members of the team. While entire UPTIDER team is involved in the review of the code scripts, the bioinformaticians are tasked with the maintenance and upkeep of the entire code versioning system. GDPR general data protection regulation, HBM human body materials, LIMS: lab information management system, NGS next-generation sequencing, UPTIDER UZ/KU Leuven Programme for post-mortem Tissue Donation to Enhance Research. Created with biorender.com.

Raw data such as sequencing outputs and digitalized histopathological tissue sections, or processed data resulting from downstream analyses are stored in a GDPR protected archival data warehouse in distinct locations (Fig. 6a). This warehouse provides a secure environment with protected role-based access and MFA. Regular backups help prevent data loss. Currently, our data occupies approximately 35 terabytes of storage within the warehouse. For frequently accessed data, we use an active data warehouse to increase transfer speed, but it does not provide backups. Transfer between the storage solutions are achieved by Globus^37^ which enables secure data transfer and tracking in the background (Fig. 6a). The link between clinical data, sample data and raw data is collected in a live Excel 365 document (Supplementary Table S5). This document allows for concurrent access and ensures that each team member accesses the most up-to-date information regarding the samples. This file also gets a backup in a separate location for data recovery. Collectively, the LIMS, storage solutions, and the live document facilitate storage and access of samples and sample data, ensuring research integrity and transparency.

### Developing and maintaining a distributed code versioning system

The distributed code versioning system has two key objectives: (i) ensuring the robustness of the 3 pillars to maintain research quality, and (ii) facilitating methodical downstream analyses of our data. We hosted an open-sourced system for the entire laboratory, allowing everyone in the team to access and track code^6,38^. The repository was structured around 4 elements to host both project specific code scripts, often maintained by one individual, and common scripts, maintained by multiple users that could easily be reused in various projects: (i) scripts for each scientific project directory, (ii) scripts and functions for QCs within the OSE, (iii) minimal code scripts that reproduced study results and (iv) scripts within shared directories for statistical analyses and data visualisation (Fig. 6b). Here, a team of 4 bioinformaticians are involved in active development and maintenance of the system which is then cloned by the entire team. The code calls for data access follow the data file structure of the data warehouses, ensuring that the appropriate files are accessed by the code scripts for every team member on their systems (Fig. 6b). Bimonthly roundtable sessions are organised, allowing code review, continuous improvements, and compliance to the system. R scripts were developed to support various sub-studies within UPTIDER, ranging from histopathological characterization, upstream and downstream processing of sequencing data to the processing of public data from cancer data repositories for our meta-analyses^26^. To analyse data from multiple sources, more than 30 interoperable scripts were also created to facilitate data processing and analysis. Altogether, the repository hosts all team-generated scripts, minimizing the risk of code loss. It facilitates code reviews, ensures knowledge is capitalized on, and makes the source code replicable and adaptable to support our research objectives.

### Performing data curation

Data generated from UPTIDER is extensive (Fig. 1, Supplementary Fig. S1), requiring continuous efforts to ensure data consistency and accuracy. To facilitate this, the team regularly engages in eCRF and LIMS days for collective data curation of clinical and sample data and metadata. This effort is assisted by more than 30 automated QC, implemented as R scripts (Fig. 6b), on eCRF and LIMS data APIs) (Figs. 4, 5) which identifies metadata features that are either missing or incorrect according to pre-established QC rules (Supplementary Table S6). These rules co-exist with the previously mentioned internal QCs of the eCRF and LIMS. In this manner, we ensure our data and metadata are always accurate (Figs. 4, 5).

### Enabling internal data reporting

To complement data curation and downstream analyses, we developed R Shiny apps^39^ that use the API export of the eCRF and the LIMS (Figs. 4, 5, Supplementary Fig. S4, S5). These apps helped visualize the status of clinical and sample metadata features in a user-friendly and interactive web environment. For instance, the eCRF dashboards enabled the compilation of essential clinical features by querying all patient records for metadata, including molecular subtype of the primary disease, treatments received during patient life, imaging modalities and metastatic lesions detected at various tissue sites (Supplementary Fig. S4). Similarly, the LIMS tool, which relies on complex queries, enabled the visualisation of the number of mirrored samples generated per patient across different sample conditions and timepoints (Supplementary Fig. S5). These tools help data curation and allow data exploration to develop research hypotheses and collaborations.

### Training of the team

Maintaining the integrity of an OSE requires active effort, communication and training^2,5^. This is especially true for UPTIDER, where objectives keep evolving, and where personnel from multiple scientific areas of expertise need to interact with the environment. In this regard, regular sessions are organised by bioinformaticians to train the team in the latest changes to the OSE and to document new requirements from the team. For instance, in the eCRF, new features were added after the team was trained to document information regarding the cause of death. Considering the rapidly evolving field of liquid biopsies and biomarker detection, new protocols were developed, and metadata structures were adapted accordingly to improve annotation and complement protocol requirements. For the code versioning system, training sessions were organized to introduce new personnel to the code repository and to the scripts they will need to access. For data inventory, trainings were conducted to optimally capture the required metadata for efficient bio-tracking. These training sessions ensure accountability in data collection and reporting, foster confident engagement, which in turn improve the integrity of the OSE.

### Performing data minimization and deposit

After the research manuscript has been peer-reviewed and accepted for publication, data is shared (Figs. 2, 3). Here, data and code minimization are performed to comply with the FAIR principles and GDPR, meaning only the essential parts necessary to reproduce the results are shared. In case of sensitive clinical data concerning patient information, a synthetic version of the minimised dataset is provided to prevent patient identification. The creation of interoperable code scripts within our code repository simplified code minimization. These scripts are also tested in a simulated environment on the code repository (Fig. 6b) that mimicked a code capsule environment where only minimal datasets are made available. Multiple local code re-runs are performed to ensure precise reproduction of code. Later, Code Ocean is used to deposit code and supporting metadata in a secured and installation-free, container-based environment^12^. Indeed, the environment outlines all the coding frameworks and supporting package libraries used to support the reproduction of code output reflecting the library versions mentioned in the research methods of the study. Moreover, the design and structure of the capsule make it convenient to export the data and environment for offline reproduction under the no lock-in philosophy of Code Ocean. During the submission of the research manuscript, code is also made available for peer-review prior to publishing the code capsule^40^. After the manuscript has been revised following peer-review, the open-access capsule is published with an accompanying DOI. The capsule also contains adequate supporting information regarding the published work and information for correspondence.

We apply an MIT license, which is a permissive license to use, modify and distribute our code with minimal restrictions^41^. We license the supporting data and metadata uploaded to the code capsule with a Creative Commons Attribution-NonCommerical-NoDerivs license (CC BY-NC-ND), to facilitate the utility of these datasets in an academic setting. As of date, 5 licensed minimized datasets and code capsules with an active DOI have been published^32,42–45^ from the UPTIDER study (Code availability), with 4 additional sub-studies under active development.

For all our studies where sequencing data is used, we upload the raw sequencing files to the European Genome-Phenome Archive (EGA) under protected access. EGA is a domain-specific trustworthy data repository where biomedical research data can be made publicly available^46^. Access to this data can be requested by external academic collaborators via a data access committee (DAC) on the EGA portal. Thereafter, following an approval from the UZ Leuven ethics committee, a data transfer agreement needs to be signed prior to accessing our sequencing data^25,47^. For access to samples from our biobank, following correspondence with the team, it requires approval by our ethics committee and a signed material transfer agreement^25,47^. These agreements ensure that the research investigation aligns with responsible secondary use of patient material and data, while also complying with ethical requirements from UZ Leuven and GDPR^25^. These conditions are effective only if the patient consented to share their tissue samples and derived data in their informed consent form, which was recorded in the UPTIDER eCRF at study inclusion.

### OA publishing

OA publishing remains the quickest way to communicate your research to a global audience while benefiting from increased visibility, discovery and reporting^16^. Most OA journals are also peer-reviewed, ascertaining the work quality. We opted for gold and diamond OA publishing as they enable unlimited public access to the research work upon publication^15^. Our commitment to OA publishing is in line with the mandates of federal funding agencies, making our work visible, accountable and transparent. This in turn, enables better research and collaboration, improving public health outcomes. As such, all research and/or review articles containing data derived from the UPTIDER study have been published in either a gold or a diamond OA journal^32,42,45,48–50^. Furthermore, protocols used to achieve our outputs are fully detailed in our publications for peer-replication. As team contributions are well documented in our publications, respective team members remain available for further correspondence.

### Dissemination and communication

Sharing the experience of building and maintaining an OSE to yield high-quality research outputs could provide a much-needed incentive for other researchers to develop their own. As open science principles are gaining traction, calls for implementation at the institutional level are increasing^4,13^. To this end, we presented our OSE internally, at open science conferences and cancer congresses to raise awareness on the benefits and the advantages of investing in an OSE for translational research^51,52^. We also advised and guided other labs that were interested in customizing an OSE to their research requirements. Expanding this, we also provide a list of software and infrastructural components used within our OSE to help researchers identify the appropriate tools for their own use (Supplementary Table S7).

### Designing a data management plan

For effective research, a DMP is generally mandatory at the project level. It provides the necessary information concerning the acquisition, consumption, storage and re-use of research data to support translational research objectives, while complying with ethical and regulatory frameworks. A DMP also contains information on personnel responsible for correspondence of study progress and the expected costs for data processing and study publication. Here, prior to developing project-specific DMPs and designing the components of the UPTIDER OSE, we designed an overarching DMP at the laboratory level, to be compatible with open science, GDPR and FAIR principles while promoting sustainable translational research in metastatic breast cancer. Building an overarching DMP helped keep a global overview of the data workflow. It also contributed to proper environment design and the generation of the mandatory project-specific DMPs. More importantly, the DMPs are active and dynamic documents that keep evolving with the growing needs of each research study.

### Assessment and continuous improvement

To sustain an evolving OSE, it is important to implement (self)-assessment strategies to identify strengths and weaknesses. We leveraged our institution’s commitment to high-quality RDM services, including a self-assessment methodology based on published models^53,54^. This tool evaluates the OSE in terms of research planning, data collection, processing, and storage. Based on the assessment outcomes, RDM support staff provide guidance and feedback. We participated in the pilot phase of the RDM assessment, evaluating our OSE’s compatibility with open science principles (Supplementary Fig. S6). After the session, we implemented improvement suggestions and scheduled yearly assessments for continuous improvement, ensuring our environment remains compatible with open science standards and evolves with the UPTIDER project needs.

## Discussion

Our OSE was built on an end-to-end approach to accommodate and sustain our continuously evolving goals in translational metastatic breast cancer research by using samples collected from UPTIDER (NCT04531696)^32^. To our knowledge, it is the most extensively documented OSE in translational research for metastatic breast cancer. Our work also presents several unique features. With annotation of more than 750 clinical features per patient, and more than 100 features per sample by multidisciplinary team members across various data types, we have helped create a comprehensive repertoire of metadata on metastatic breast cancer samples. Complementing this, sample and data storage in an institutional biobank and data warehouses enables long-term accessibility to both the team and the researchers willing to use our data. By integrating multiple sources of information such as sequencing data, digitalized tissue sections, sample metadata and clinical data, our OSE enables reproducible and extensive investigations into the biology of the metastatic disease in breast cancer, which is currently an unmet need.

**Fig. 7:**
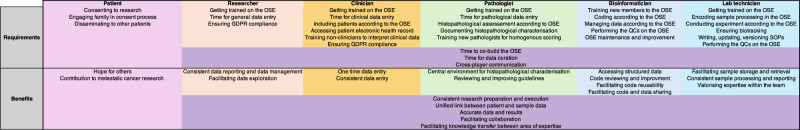
Essential OSE takeaways per player. Figure describes the key takeaways within the OSE to improve generalizability and utility for each player in a translational research team. Takeaways are divided into requirements and benefits to highlight prerequisites for successful implementation of the OSE and the long-term benefits derived from this implementation. Players are represented by their colour as reflected in Fig. 1. FAIR: findable, accessible, interoperable and reusable, OSE: open science environment, QC: quality checks, SOP: standard operating protocol. Created with Microsoft Excel.

By implementing and maintaining open science standards for data collection and sharing, our environment makes our research accessible and reproducible. Additionally, with the help of internal QC, eCRF and LIMS days, data compliance with regulatory frameworks is sustained. While open science principles are often reduced to code and data sharing with OA publishing, our work offers perspectives into the utility and benefits of building an OSE from study conception. Our OSE included regular trainings, peer-dissemination, collaboration with the RDM network, CTC, biobank, internal data reporting, and a self-assessment procedure. Key takeaways for each player are summarized in Fig. 7 to incentivize others to build on our work. Our OSE is complementary to existing open science efforts such as the open science framework (OSF) and the tranSMART project^55,56^. These provide infrastructural support to facilitate registered research, data integration, data management, hypothesis testing, data sharing, collaboration and reproducibility in a secure online environment. While the utility of the OSF was shown in the Reproducibility Project: Cancer Biology^57–60^, these platforms are however, not designed to manage day-to-day open science research^61^. These initiatives, along with our environment, are in line with ongoing national and international open science efforts such as the National Pandemics Cohorts Network and the European health data space^36,62^. These also implement a coordinated OSE for harmonised patient consent, study protocol registration, biobank data collection, data processing, and data sharing.

In our perspective, the implementation of an OSE starts by a complete study design alongside an overarching DMP, making sure all requirements of a research study are identified. Additionally, for continuous commitment to open science, a motivated team is crucial as time and efforts are needed for trainings and for contributing to the OSE. Compliance with the OSE is ensured through regular meetings, highlighting the importance of open science during the recruitment of new team members, and adding open science objectives to job descriptions and PhD proposals. To ensure ethical compliance in translational research data management, adequate human resources are essential, as sharing sensitive information like patient EHR and tissue samples are protected by regulations^7,10,24,25^. While mandatory, they can also increase administrative and legal burden, delaying access to sample and data collection for research collaboration. Most importantly, it also necessitates experienced researchers in the team, whose appointments are rarely covered by research grants, increasing burden on project timelines. Besides this, maintaining an OSE with regular sample collection, processing, maintenance of LIMS and eCRF paired with costs for long-term storage and open access publishing can be costly and logistically intense. Obtaining the required constant funding is challenging, since many research funding applications do not fully cover these costs. For example, many open-access journals have extremely high APC which unfortunately, are above the category limits approved by the funding agencies, increasing financial burden. This is leading many researchers to look for auxiliary funding and institutional support, which may or may not be fruitful^63^. Consequently, preprints and projects like the PCI are gaining traction to freely access a manuscript alongside free peer-review process after preprint deposit^18^. While open science practices are desired by everyone, lack of proper encouragement, recognition and appreciation for researchers who invested time and resources to implement open science practices and build an OSE, results in diminished incentives for others to adopt similar practices. For long-term implementation of sustainable open science research practices, an institutional network with active training, guidance and communication is needed to help support researchers^2,19,27^. This is also highlighted in the recent publication by the League of European Research Universities which provides insights into initiatives by europe to promote open science practices in academia (https://www.leru.org/publications/open-science-and-its-role-in-universities-a-roadmap-for-cultural-change) and european research initiatives to provide training opportunities to implement open and FAIR science^64^. Considering this, we provide a checklist of good practices to facilitate the implementation of an OSE irrespective of the level of resources, using institutional collaborative tools (Supplementary Table S8).

With increasing complexity of biomedical and clinical data, reproducibility of results remains a key obstacle for progress and collaboration within translational research. This is especially important in metastatic cancer, as prediction models and artificial intelligence (AI) are increasingly being used to incorporate health data with genomic data in translational research^26,33,65^. These tools can help in unsupervised detection of new biomarkers to stratify patients based on treatment response or to predict clinical outcomes at diagnosis, enhancing precision medicine and disease management^66,67^. However, limited and/or incomplete data structures can undermine model efficacy, increasing the risk of misinformed findings^68^. Hence, a large amount of compatible, well-structured and accurate patient data analysed using reproducible code are crucial to effectively implement AI^26,69,70^. This can be facilitated by an OSE and enhancing translational research outputs^33^. Within our OSE, we have created optimized data and metadata structures for programmatic querying through APIs for all features available in the eCRF, the LIMS, and associated sequencing files in both raw and processed forms. This provides the necessary groundwork to implement AI query-based modelling within our OSE, allowing to incorporate multi-modal data structures. In the future, with growing patient inclusion, the environment can assist with precision model design which could improve diagnostic accuracy, precision medicine and biomarker-based risk-assessment^66,71,72^. In this regard, we believe that our environmental design is well-suited and places us at the forefront of a new era for translational research in metastatic breast cancer.

Thus, by taking our experiences into account, we encourage consideration in investing and designing an OSE that could have potential long-term benefits for translational and public health research. While our environment was tailored to the needs of UPTIDER, we believe our design could be generalized and independently customized. This way, we help a new generation of translational researchers build their own open science environments to promote research integrity and data sharing.

## Methods

### Post-mortem tissue donation programme

UPTIDER (UZ/KU Leuven Programme for post-mortem Tissue Donation to Enhance Research) is a monocentric tissue donation programme that enrols consenting patients with metastatic breast cancer (NCT04531696). UPTIDER received approval from the ethical committee of Universitair Ziekenhuis Leuven (S64410, approved November 30, 2020). Written informed consent was obtained from all participants and all relevant ethical regulations, including the Declaration of Helsinki, were complied with. Additional information regarding patient inclusion, sample collection and data curation of patient information study design can be consulted from is present in our original publication^32^. The core UPTIDER team consists of medical, surgical and gynaecological oncologists, expert breast pathologists, anaesthesiologists, bioinformaticians, biostatisticians, translational research managers, fundamental biomedical scientists, lab scientists and technicians. This core team is also supported by expert personnel from forensic medicine and radiology.

### Environment design

The key objective of the UPTIDER OSE is to promote sample and data sharing while sustaining collaboration with multidisciplinary stakeholders involved in translational research on metastatic breast cancer. In this context, the 4 following pillars were designed to support and sustain the long-term utility of the OSE: (i) Clinical data, (ii) Sample data, (iii) Storage and (iv) Code versioning (Fig. 2a). These pillars were designed to be prospectively flexible and adaptable to adapt and evolve to the prospective requirements of UPTIDER. The design of these 4 pillars incorporated 3 crucial principles: (i) FAIR principles making it convenient for multidisciplinary stakeholders and team members to reuse available UPTIDER data and assist with long-term sharing, (ii) GDPR and belgian act on experiments on human beings (7 May 2004, Belgian Clinical Trials Directive), to account for pseudonymization of sensitive patient data before inclusion for prospective regulated data collection, data reuse, and pseudonymization of data sets prior to sharing, (iii) Collection of samples and sample data in line with good clinical practice (GCP) guidelines allowing for annotation of metadata and long-term data storage while satisfying the requirements for FAIR, GDPR and the Belgian Clinical Trials Directive (Fig. 2a). Of note, GDPR does not apply to deceased patients, but the study still complies with it as a general research principle to safeguard the privacy of the patients and their families.

### Clinical data collection

Collection of clinical data from patient EHR was performed using an eCRF^73^ based on REDCap (v15.0.37) to corroborate translational findings with clinical disease progression, treatment regimens, and patient quality of life. An eCRF is an electronic document designed to record and collect elements from the patient EHR to address a specific research question while maintaining data transparency and privacy. While eCRF is conventionally used to record interventional clinical trial data, it was used here to facilitate data collection in a protected environment after receiving patient consent for the use of EHR data. Although principles exist to guide the structure of the eCRF, the specific feature selection is highly dependent on the project^74^. Consequently, the entire team contributed to identifying the necessary features to meet the objectives of UPTIDER. With security protocols such as role-based access, MFA and data viewing authorization, an eCRF can be used to extract and document authorised clinical information without cloning the entire patient EHR (Environment design, Methods). For clear identification and documentation, each patient eCRF was pseudonymised and given a non-descriptive patient identifier. Moreover, recording and reporting was performed under the guiding principles of GDPR with a provision for data validation and audit trail. The eCRF was also designed to use SQL-based queries to populate certain fields. Of note, the eCRF is hosted on a secure institutional server that is actively maintained and backed up by a specialized team from the institution. REDCap offers both a web-based graphical user interface (GUI) and an API allowing programmatic access to the database.

### Metadata collection

Collection of data and metadata from samples (solid tissue/liquid biopsy) acquired after patient inclusion (pre-mortem/post-mortem) into the study was performed using a LIMS based on Labcollector (v6.262). LIMS are built on open data exchange standards to enable data sharing and interoperability in compliance with the FAIR principles^9^. A LIMS enables the structured documentation and update of metadata according to standardized data templates. By providing a unique non-descriptive identifier to each registered sample, the LIMS helps in accessing and tracking the sample throughout its existence. Metadata curation and LIMS templates were designed according to the standard preanalytical coding for biospecimens (SPREC)^75^ requirements of our institutional biobank. In-built ELN also enabled documentation of downstream sample processing using SOP templates and versioning with protocol amendments^32^. The LIMS, like the eCRF, is also hosted on a secured and backed-up server that offers access both via a web-based GUI and an API.

### Sample and data deposit

Identifying suitable storage locations was essential for securely storing collected samples, derived data and results. This eventually facilitates long-term deposit, access and sharing. The institutional biobank was used to deposit study samples, while data warehouses (active and archival) were used to deposit derived sample data and study results. These institutional data warehouses are protected with MFA and role-restricted access, enabling secure deposit of derived sample data and convenient real-time retrieval. The archival storage offers a mirror copy stored in a different geographic location, enabling data recovery. Additionally, the data structure and file hierarchy present on the archival storage is replicated on the computing systems of each team member to enable data access and code sharing.

### Code version control

A distributed code versioning system was used to create, document, track, review and share code modules amongst the team members^38^. This open-source system relies on FramaGit (https://framagit.org) and was designed to host code with parallel access to multiple projects and users. The structure relies on 4 components: (i) code for scientific projects including downstream analyses of derived sample data, (ii) code for supporting the OSE and to perform quality checks (QC) on LIMS and eCRF metadata, (iii) code used to design a local capsules to simulate and reproduce research results for study publication with minimal datasets to publish a code capsule using CodeOcean^12,40^ after each research publication and (iv) reusable code as part of shared directories. R (>4.0) was chosen as the preferred software language for downstream data processing, curation, analyses and code sharing. The code versioning system was implemented to allow standardization and review of the code among bioinformaticians, while allowing non-bioinformaticians to be able to run the code after proper training.

### Environment timelines

The environment has two serial timelines: Research Phase and Publication Phase (Fig. 2b). The research phase begins at patient inclusion into the study till post-mortem tissue donation, which is then followed by downstream analysis of data derived from samples along with data QC. Within this phase, data is collected and annotated according to the FAIR principles. The publication phase begins right after downstream data analysis until study publication, which is then followed by online data deposit, OA publication and data sharing. This phase is crucial for data and code sharing in a GDPR compliant manner.

## Supplementary information

Supplementary Information

Supplementary Table S1

Supplementary Table S2

Supplementary Table S3

Supplementary Table S4

Supplementary Table S5

Supplementary Table S6

Supplementary Table S7

Supplementary Table S8

## Data Availability

All supporting information regarding the methodology and the implementation of online tools have been provided as Supplementary Tables and Figures.

## Code availability

All information regarding the code capsule published at the laboratory for translational breast cancer research can be found at www.ltbcr.be. The code capsules created and published for UPTIDER sub-studies can be consulted here: 10.24433/CO.6936341.v1, 10.24433/CO.0195636.v1, 10.24433/CO.4977615.v1, 10.24433/CO.8724699.v1, 10.24433/CO.6616975.v1. 10.24433/CO.6936341.v1, 10.24433/CO.6936341.v1, 10.24433/CO.6936341.v1, 10.24433/CO.6936341.v1.
