# Supplementary material for: Enhancing translational research in metastatic cancer through an open science environment: the UPTIDER experience
Source: NPJ Precis Oncol. 2025 Nov 6;9:341. doi: 10.1038/s41698-025-01110-5 (PMC12592526; doi:10.1038/s41698-025-01110-5)
Supplement: Supplementary file 1 — Supplementary Information [file 41698_2025_1110_MOESM1_ESM.pdf]

Supplementary Information

Enhancing translational research in metastatic cancer through an open science environment: the UPTIDER experience

Pabba, et al.

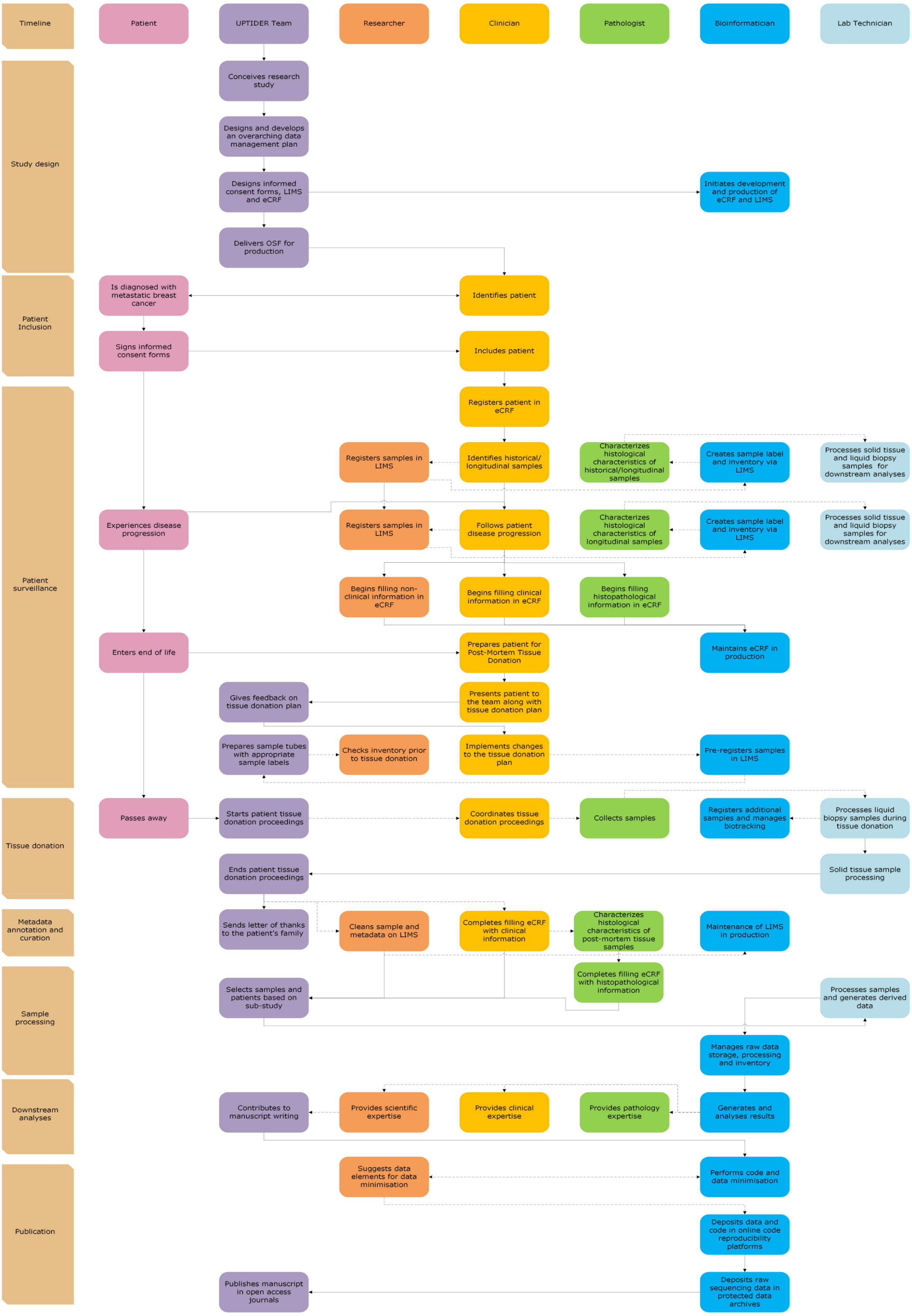
Timeline
Patient
UPTIDER Team
Researcher
Clinician
Pathologist
Bioinformatician
Lab Technician
Study design
Conceives research study
Designs and develops an overarching data management plan
Designs informed consent forms, LIMS and eCRF
Initiates development and production of eCRF and LIMS
Delivers OSF for production
Patient Inclusion
Is diagnosed with metastatic breast cancer
Identifies patient
Signs informed consent forms
Includes patient
Patient surveillance
Registers patient in eCRF
Registers samples in LIMS
Identifies historical/ longitudinal samples
Characterizes histological characteristics of historical/longitudinal samples
Creates sample label and inventory via LIMS
Processes solid tissue and liquid biopsy samples for downstream analyses
Experiences disease progression
Registers samples in LIMS
Follows patient disease progression
Characterizes histological characteristics of longitudinal samples
Creates sample label and inventory via LIMS
Processes solid tissue and liquid biopsy samples for downstream analyses
Begins filling non-clinical information in eCRF
Begins filling clinical information in eCRF
Begins filling histopathological information in eCRF
Enters end of life
Prepares patient for Post-Mortem Tissue Donation
Maintains eCRF in production
Gives feedback on tissue donation plan
Presents patient to the team along with tissue donation plan
Prepares sample tubes with appropriate sample labels
Checks inventory prior to tissue donation
Implements changes to the tissue donation plan
Pre-registers samples in LIMS
Tissue donation
Passes away
Starts patient tissue donation proceedings
Coordinates tissue donation proceedings
Collects samples
Registers additional samples and manages biotracking
Processes liquid biopsy samples during tissue donation
Ends patient tissue donation proceedings
Solid tissue sample processing
Metadata annotation and curation
Sends letter of thanks to the patient's family
Cleans sample and metadata on LIMS
Completes filling eCRF with clinical information
Characterizes histological characteristics of post-mortem tissue samples
Maintenance of LIMS in production
Completes filling eCRF with histopathological information
Sample processing
Selects samples and patients based on sub-study
Processes samples and generates derived data
Manages raw data storage, processing and inventory
Downstream analyses
Contributes to manuscript writing
Provides scientific expertise
Provides clinical expertise
Provides pathology expertise
Generates and analyses results
Publication
Suggests data elements for data minimisation
Performs code and data minimisation
Deposits data and code in online code reproducibility platforms
Publishes manuscript in open access journals
Deposits raw sequencing data in protected data archives

Supplementary Figure S1. **The UPTIDER process from study design to patient inclusion and study publication, extended from Figure 2.** A flowchart and timeline describe the key processes within UPTIDER attributed to each of the players in the team. Here, additional actions prior to patient inclusions and during patient surveillance have been described here. eCRF: electronic case report form, LIMS: lab information management system, OSE: open science environment, UPTIDER: UZ/KU Leuven Post-mortem Tissue Donation to Enhance Research. Created with Microsoft Visio.

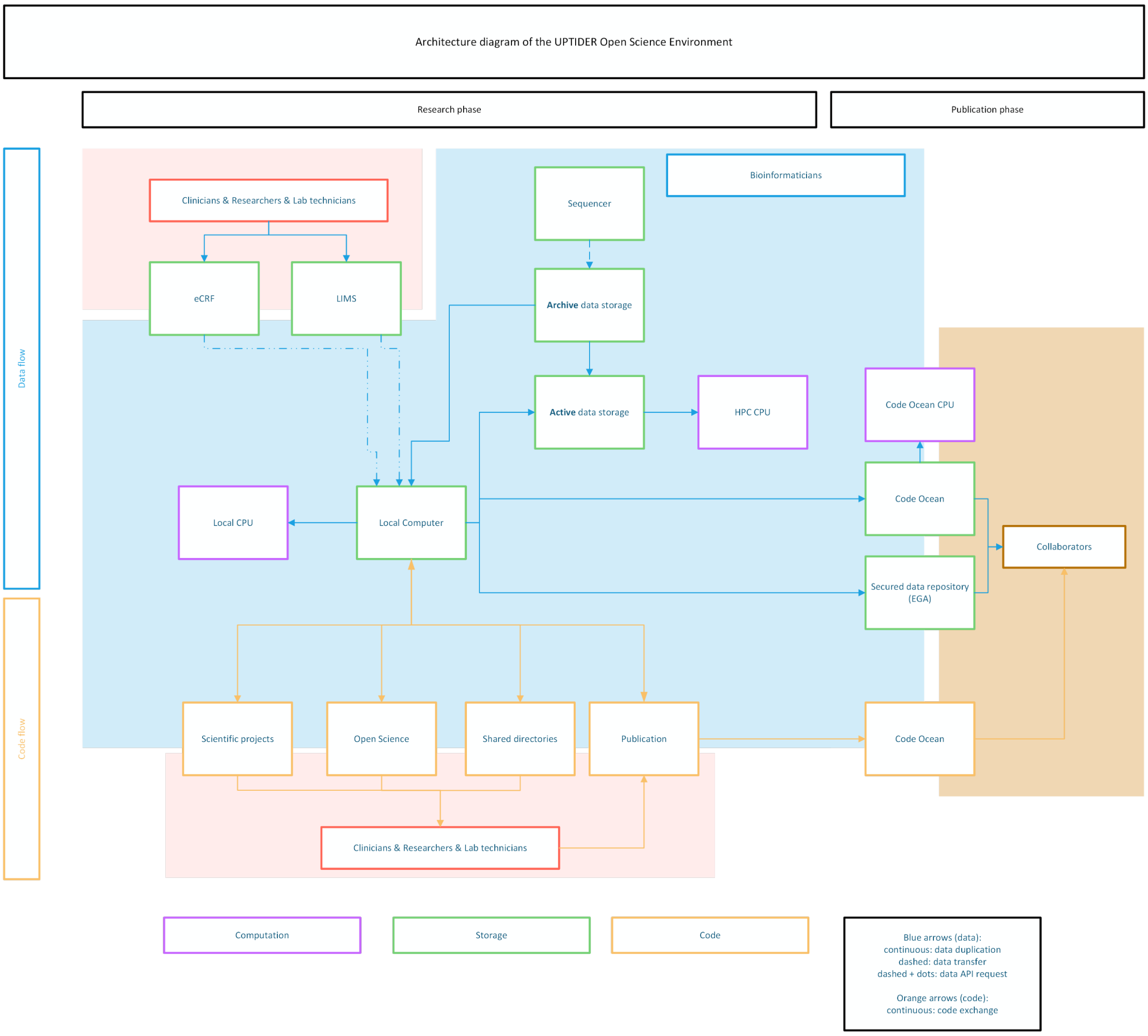

Supplementary Figure S2. **The architecture diagram of the UPTIDER OSE.** A flowchart describes the key interactions between logistical units: computation, storage and team players within the OSE, highlighting data and code flow. Here, arrows outline flow of data and code between players and tools between research and publication phases. Additionally, computation has been used to reflect the tool-based actions for data and code computation.

Team players have also been re-classified into three key groups (bioinformaticians, non-bioinformaticians and collaborators) to provide a simplified architecture. Arrow type reflects actions such as duplication, transfer and API request. API: application programming interface, CPU: central processing unit, eCRF: electronic case report form, EGA: European genome-phenome archive, HPC: high performance computing, LIMS: lab information management system, UPTIDER: UZ/KU Leuven Post-mortem Tissue Donation to Enhance Research. Created with Microsoft Visio.

a

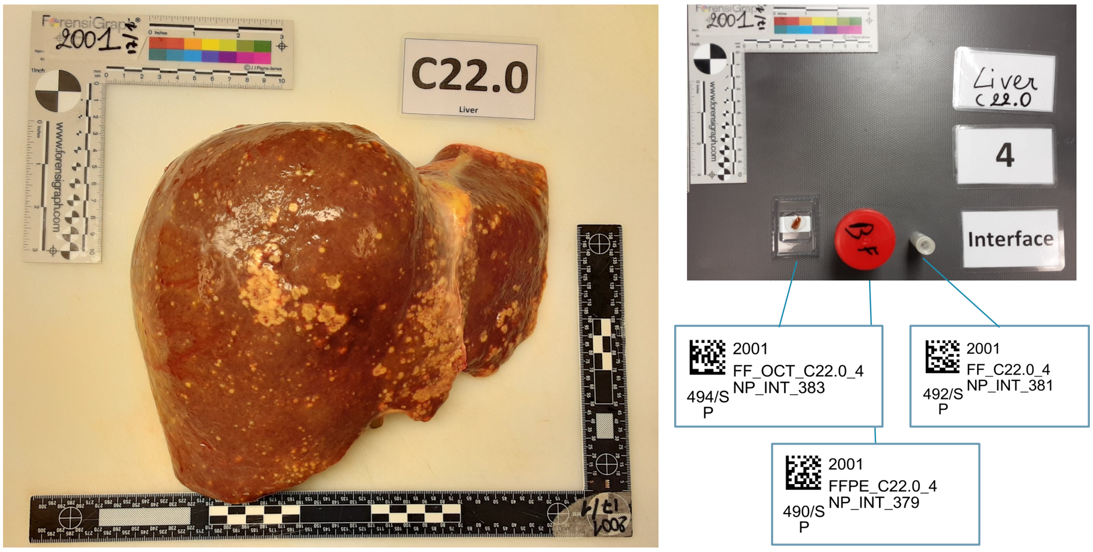

b

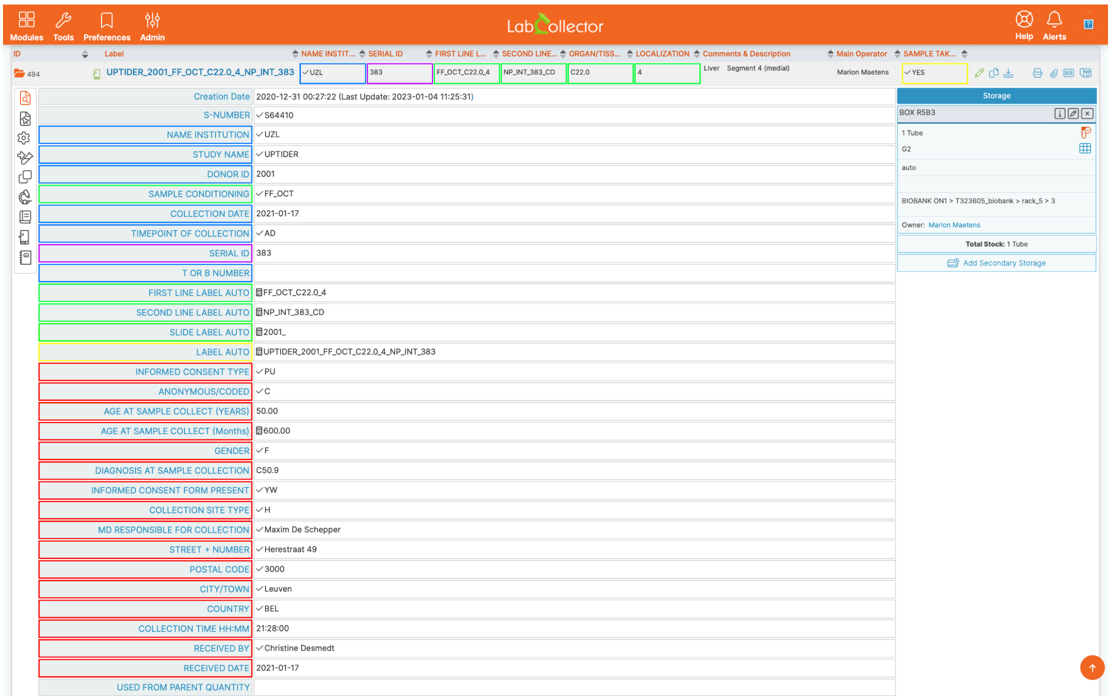

Supplementary Figure S3. **An example of the LIMS metadata.** a. On the left is a liver (ICD-O-3 code: C22.0) from patient 2001 that was completely invaded with a diffused tumour pattern seen at autopsy during tissue donation. On the right are metastases collected from the tumour interface of segment 4 of the liver using our mirrored sample approach. The sample labels give information on key metadata such as sample conditioning, organ location, patient ID, sample ID and LIMS ID along with a QR code for metadata collection and revision on LIMS. b. Completed metadata of the same metastasis on a pre-registered LIMS record after sample collection at the autopsy. FF: fresh frozen, FF-OCT: fresh frozen in OCT medium, FFPE: formalin fixed paraffin embedded, INT: interface, NP: non-pathologic. Schematic based on Labcollector (v6.262).

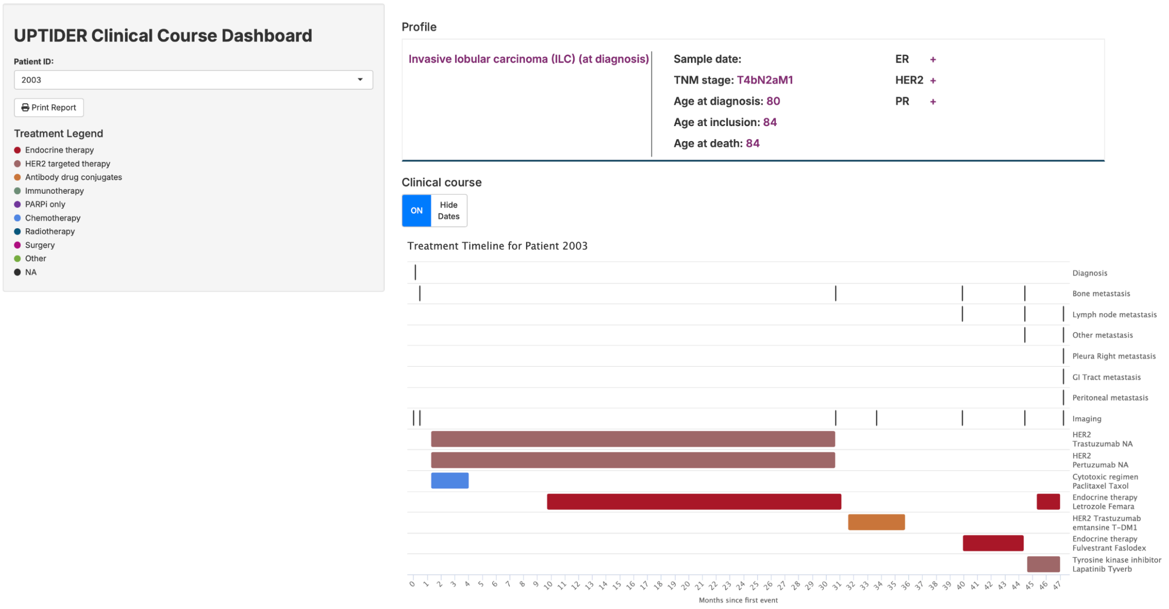

Supplementary Figure S4. **An example of the eCRF dashboard**. The dashboard displays the clinical disease course of patient 2003 starting from the diagnosis of the primary tumour. Key clinical characteristics including age at diagnosis, inclusion and death, date of sampling, tumour staging, status of tumour biomarkers and tumour histology at primary diagnosis, along with incidence of various metastases and duration of various treatments are also mentioned here. +: positive, ER: estrogen receptor, GI: gastrointestinal, HER2: human epidermal growth factor receptor 2, PR: progesterone receptor, T-DM1: trastuzumab emtansine, TNM: tumour, node and metastasis staging. Created using RShiny. Schematic based on REDCap (v15.0.37).

a

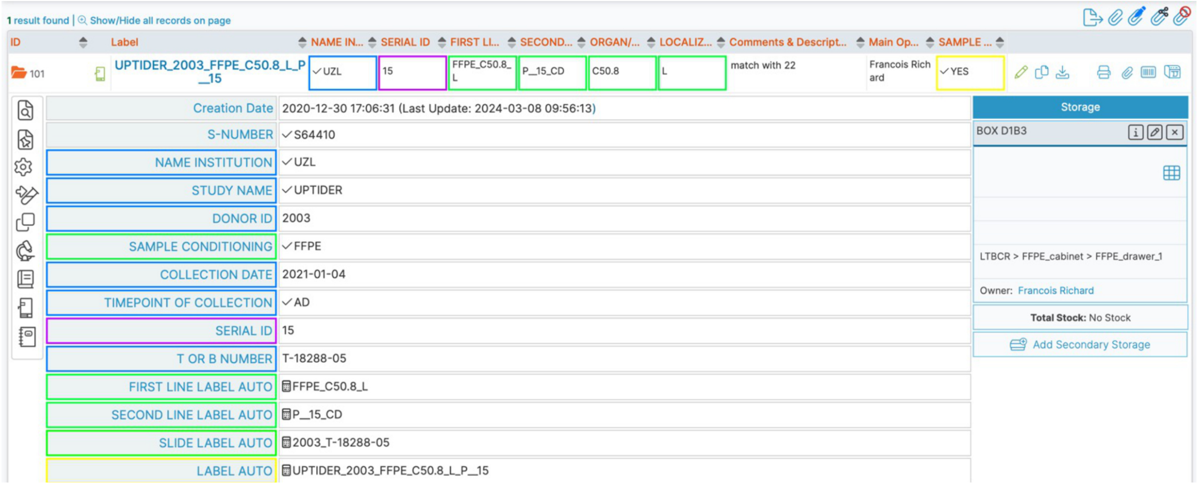

b

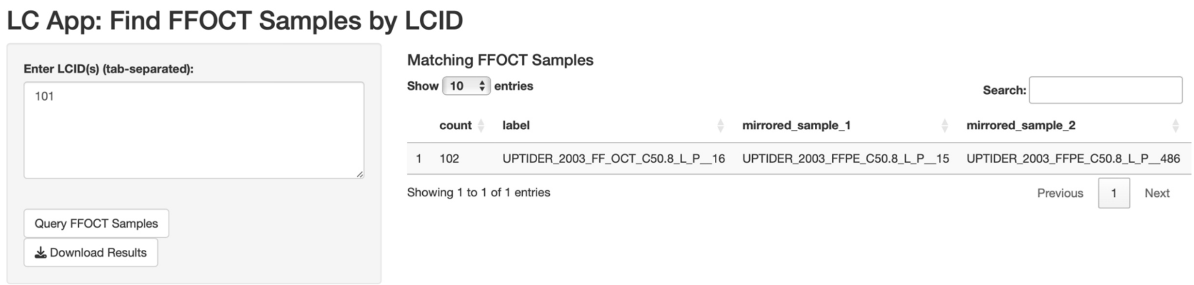

Supplementary Figure S5. **An example of the LIMS tool.** a. A LIMS record of a breast (ICD-O-3 code: C50.8) FFPE mirrored sample collected at autopsy during tissue donation of patient 2003, along with all the metadata linked to the sample. b. LIMS tool identifying the appropriate FF-OCT mirrored sample linked to the FFPE sample mentioned above, which will be used for library preparation prior to next-generation DNA or RNA sequencing. Mirrored sample 1 represents the closest FFPE link to any FF-OCT sample within the LIMS database, with successive mirrored samples (2, 3, 4...) as possible links based on the available metadata. FF-OCT: fresh frozen in optimal cut temperature medium, FFPE: formalin fixed paraffin embedded, ID/LCID: LIMS ID, L: left, LIMS: lab information management system, P: pathologic. Created using RShiny. Schematic based on Labcollector (v6.262).

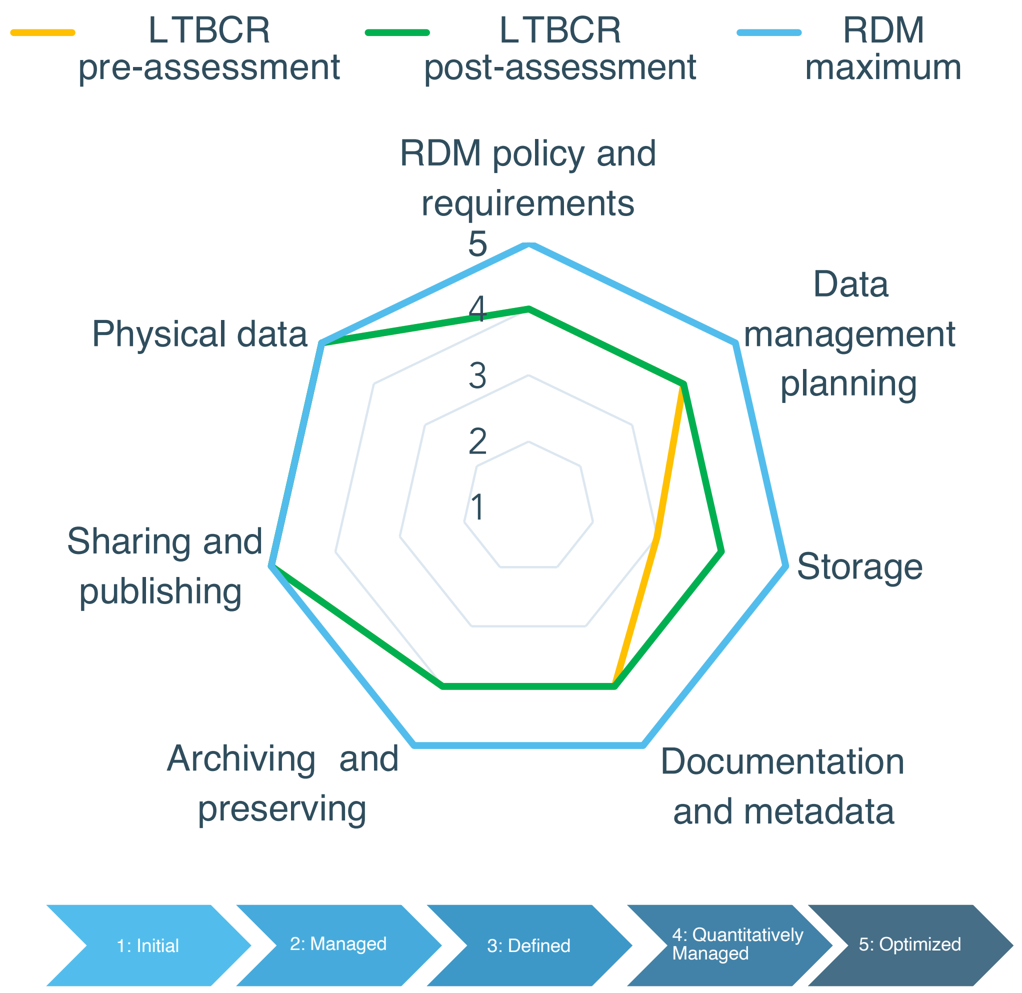

Supplementary Figure S6. **Results of the RDM assessment.** A radar plot describes the performance of our open science environment standards before and after the first assessment session (yellow and green) when compared to the reference standard set by our institutional research data management (blue). The scores reflect the status of our environment across 5 levels from initial to optimized. This assessment rated our environment to be between advanced and optimized across 7 different categories. LTBCR: laboratory for translational breast cancer research, RDM: research data management. Created with Microsoft powerpoint.

Supplementary Data 1: Supplementary Table S1 (eCRF founder document)

Supplementary Data 2: Supplementary Table S2 (eCRF codebook)

Supplementary Data 3: Supplementary Table S3 (LIMS founder document)

Supplementary Data 4: Supplementary Table S4 (LIMS codebook)

Supplementary Data 5: Supplementary Table S5 (Bridge document biobank to data warehouse)

Supplementary Data 6: Supplementary Table S6 (LIMS QC)

Supplementary Data 7: Supplementary Table S7 (Software components and versions)

Supplementary Data 8: Supplementary Table S8 (Good practices checklist)
